# Determinants of sedentary behavior in community-dwelling older adults with type 2 diabetes based on the behavioral change wheel: a path analysis

**DOI:** 10.1186/s12877-024-05076-0

**Published:** 2024-06-06

**Authors:** Xiaoyan Zhang, Dan Yang, Jiayin Luo, Meiqi Meng, Sihan Chen, Xuejing Li, Yiyi Yin, Yufang Hao, Chao Sun

**Affiliations:** 1 Department of Vascular Surgery, Beijing Hospital, National Center of Gerontology, Institute of Geriatric Medicine, Chinese Academy of Medical Sciences, Beijing, P.R. China; 2 School of Nursing, Beijing University of Chinese Medicine, No. 11, Beisanhuandonglu, Chaoyang District, Beijing, People’s Republic of China; 3 Department of Nursing, Beijing Hospital, National Center of Gerontology, Institute of Geriatric Medicine, Chinese Academy of Medical Sciences, Beijing, P.R. China

**Keywords:** Type 2 diabetes, Sedentary behavior, Older adults, Community, Path analysis, Behavior change wheel, Determinants

## Abstract

**Background:**

Sedentary behavior (SB) is deeply ingrained in the daily lives of community-dwelling older adults with type 2 diabetes mellitus (T2DM). However, the specific underlying mechanisms of the determinants associated with SB remain elusive. We aimed to explore the determinants of SB based on the behavior change wheel framework as well as a literature review.

**Methods:**

This cross-sectional study recruited 489 community-dwelling older adults with T2DM in Jinan City, Shandong Province, China. Convenience sampling was used to select participants from relevant communities. This study used the Measure of Older Adults’ Sedentary Time-T2DM, the Abbreviated-Neighborhood Environment Walkability Scale, the Social Support Rating Scale, the Lubben Social Network Scale 6, the Subjective Social Norms Questionnaire for Sedentary Behavior, the Functional Activities Questionnaire, the Numerical Rating Scale, the Short Physical Performance Battery, and the Montreal Cognitive Assessment Text to assess the levels of and the determinants of SB. Descriptive statistical analysis and path analysis were conducted to analyze and interpret the data.

**Results:**

Pain, cognitive function, social isolation, and social support had direct and indirect effects on SB in community-dwelling older adults with T2DM (total effects: *β* = 0.426, *β* = -0.171, *β* = -0.209, and *β* = -0.128, respectively), and physical function, walking environment, and social function had direct effects on patients’ SB (total effects: *β* = -0.180, *β* = -0.163, and *β* = 0.127, respectively). All the above pathways were statistically significant (*P* < 0.05). The path analysis showed that the model had acceptable fit indices: RMSEA = 0.014, χ ^2^/df = 1.100, GFI = 0.999, AGFI = 0.980, NFI = 0.997, RFI = 0.954, IFI = 1.000, TLI = 0.996, CFI = 1.000.

**Conclusion:**

Capability (physical function, pain, and cognitive function), opportunity (social isolation, walking environment, and social support), and motivation (social function) were effective predictors of SB in community-dwelling older adults with T2DM. Deeper knowledge regarding these associations may help healthcare providers design targeted intervention strategies to decrease levels of SB in this specific population.

**Supplementary Information:**

The online version contains supplementary material available at 10.1186/s12877-024-05076-0.

## Introduction

Type 2 diabetes mellitus (T2DM) [1] is the most common subtype of diabetes and is characterized by inadequate or impaired insulin secretion. Globally, the prevalence of diabetes among older adults aged 65–99 years is 19.3%; the number of individuals with T2DM is projected to reach 195.2 million by the year 2030 [2]. Notably, China bears the highest burden of diabetes among older adults individuals worldwide; among the 35.5 million people with diabetes in China, more than 90% of them are diagnosed with T2DM [3]. Inadequate management of T2DM can give rise to complications such as nephropathy, retinopathy, neuropathy, and lower limb arteriopathy [1]. These complications exert a substantial economic and psychological burden on individuals, families, and society [1]. Consequently, there is a pressing need to prevent and manage risk factors in order to provide comprehensive care for T2DM and promote healthy aging. Sedentary behavior (SB) has emerged as a novel health risk factor for T2DM, owing to shifts in lifestyle, aging processes, and declining physical function.

SB is any waking behavior characterized by an energy expenditure is equal to or less than 1.5 metabolic equivalents (METs), while in a sitting, reclining or lying posture [4]. Notably, SB extends beyond individuals with insufficient physical activity, defined as not meeting the recommended levels of physical activity to comply with current guidelines [5]. Even individuals who meet the daily recommended levels of moderate to vigorous physical activity may still experience the detrimental effects of SB [6]. A global survey [7] showed that the average sedentary time for individuals aged 60 is 9.4 h, which accounts for 65–80% of their waking hours. A related study in China [8] revealed that in community-dwelling older adults with T2DM, the average daily sedentary time was 7.01 ± 2.15 h, with 32.6% of individuals spending ≥8 h per day engaged in SB. Moreover, a meta-analysis [9] showed a linear dose‒response relationship between sedentary time and T2DM risk, indicating a 5% increase in the T2DM risk for each additional hour of SB. Epidemiological studies have confirmed the association between SB and adverse health outcomes, including metabolic syndrome [10–12], obesity [13], cancer [14], cardiovascular disease [15, 16], and all-cause mortality [17]. It is clear that SB is a significant health risk factor for T2DM. Therefore, attention should be devoted to SB, as interventions aimed at reducing SB in T2DM patients can have a positive impact on the comprehensive management of the disease.

Few empirical studies have explored the factors contributing to SB among community-dwelling older adults with T2DM. Only a small number of studies have explored the relevant influencing factors, with a primary focus on general individual characteristics such as BMI, waist circumference, educational attainment [13, 18, 19], and cognitive function [8]. While research on the determinants of SB among community-dwelling older adults with T2DM is scant, there is much more research on SB among the general older adult population. A recent scoping review [20] identified three primary domains influencing SB among older adults: personal dimensions (e.g., physical function, cognitive ability, and pain), interpersonal dimensions (e.g., social isolation, social norms, and caregiver burden), and environmental dimensions (e.g., lack of community activities and facilities promoting standing and physical activity). Sebastien F. M. Chastin and colleagues conducted a literature analysis on SB determinants in older adults using Owen’s Ecological Model as a theoretical framework [21]. Their study emphasized that personal factors (such as age and health status) were the most frequently researched determinants, while environmental factors (such as transportation, neighborhood safety, and resting places) were less explored. Jorge et al. proposed a well-established conceptual framework, drawing from Owen’s Ecological Model of SB and literature on SB among older adults [22, 23]. This framework, referred to as the Conceptual Model of SB in Older Adults, aims to identify the factors contributing to SB in older populations. The extensive research on the determinants of SB among older adults can be a valuable reference for studying the factors influencing SB in community-dwelling older adults with T2DM. However, the mechanisms through which these determinants exert their influence have not been empirically verified. The Behaviour Change Wheel framework, a comprehensive framework for understanding behavior change, can provide a theoretical basis for elucidating the underlying mechanisms of these determinants.

The Behavior Change Wheel framework [24] was developed by Michie and her colleagues in 2011 after evaluating 19 theoretical frameworks on behavior change. It is widely recognized as a comprehensive and influential theoretical framework for health behavior research. The Behavior Change Wheel framework encompasses almost all behavioral determinants and can be used as a foundation for understanding the determinants of SB in older adults with T2DM. The Behavior Change Wheel framework primarily consists of the Capability-Opportunity-Motivation-Behavior model and the Theoretical Domain Framework. The Capability-Opportunity-Motivation-Behavior model explains behavior change by considering capabilities, opportunities, and motivation as influencing factors. The Theoretical Domain Framework [25, 26] is a specific adaptation of the Capability-Opportunity-Motivation-Behavior model and provides a detailed breakdown of its concepts (see Appendix 1).

Capability generally refers to the physical or mental capacity required to perform specific behaviors, including knowledge, skills, comprehension, and reasoning. According to the Jorge’s conceptual model and literature reviews, three key capability factors associated with SB in older adults have been identified. First, current studies have shown a significant association between decreased physical function and increased SB [27], pain is another crucial ability factor related to SB [28]. Research indicates that increased pain perception in older adults is associated with a higher frequency of SB [29]. cognitive function is one of the essential indicators for assessing the health of older adults. Studies have demonstrated that older adults who engage in less SB better cognitive function, particularly in executive functions and memory [30].

Opportunity can be physical or social factors that may encourage or enable behavior. According to the Jorge’s conceptual model and literature review, four potential opportunity factors may influence SB in older adults. Firstly, the quality of the walking environment may directly affect the occurrence of SB in older adults [21, 31]. Better walking infrastructure (e.g., good street lighting, flat sidewalks) and high levels of safety can reduce sedentary time in older adults [32]. Secondly, social support, as an essential interpersonal factor, includes guidance and encouragement from family, friends, and healthcare professionals. These are important facilitators for older adults to receive physical activity advice, including recommendations to reduce sedentary behavior [33]. Moreover, social isolation is strongly associated with increased SB in older adults. Research suggests that social isolation and high levels of loneliness may lead to significantly reduced opportunities for social and physical activities among older adults, thereby increasing sedentary time [34]. Finally, social norms also have a significant impact on sedentary behavior in older adults. Studies have shown that social norms are associated with sitting time in specific contexts (e.g., sitting while reading, sitting during hobbies, and sitting while socializing) [35].

Motivation is a complex concept that encompasses automatic cognitive processes, basic drives, and reflective cognitive processes, all of which can influence an individual’s willingness to engage in a behavior. A literature review revealed that research on motivational factors influencing SB in older adults is insufficient [36]. By matching the Jorge’s conceptual model with the Theoretical Domains Framework concepts, social function emerges as one of the key motivational factors, that is particularly important for understanding the impact on SB in older adults. Social function in older adults includes engagement and activity levels within family, friends, and community [37], which can significantly reduce sedentary time [38]. Furthermore, assuming the role of caregiving for an ill spouse can increase sedentary time, with such roles being influenced by family expectations [39].

**Fig. 1:**
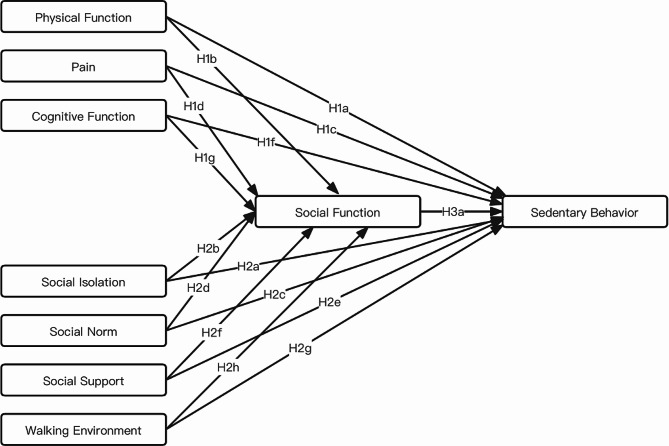
The hypothetical path model based on the BCW (COM-B and TDF) and literature review

Based on Jorge’s conceptual model of SB among older adults and an extensive literature review, we have integrated pertinent factors into both the Theoretical Domain Framework and the Capability-Opportunity-Motivation-Behavior model using the Behavior Change Wheel framework as our guiding framework. Hence, we have developed a pathway that elucidates the relationships among the determinants of SB in older adults with T2DM. These determinants include the walking environment, social isolation, social norms, social support, social function, pain, physical function, and cognitive function. This pathway forms the foundational structure for our hypothesized role model, as illustrated in Fig. 1. Based on the connotation of each domain in the Theoretical Domain Framework, their corresponding relationship with the the Capability-Opportunity-Motivation-Behavior model, and insights from previous studies, we assigned physical function, pain, and cognitive function to the “Capability” category; we assigned social function to the “Motivation” category; and we assigned social isolation, social norms, social support, and walking environment to the “opportunity” category. We combined theory and literature to develop the following hypotheses:

***Hypothesis 1***: Capability affects SB (H1a: Physical function has a direct effect on SB, H1b: Physical function has an indirect effect on SB, H1c: Pain has a direct effect on SB, H1d: Pain has an indirect effect on SB, H1f: Cognitive function has a direct effect on SB, and H1g: Cognitive function has an indirect effect on SB).

***Hypothesis 2***: Opportunity affects SB (H2a: Social isolation has a direct effect on SB, H2b: Social isolation has an indirect effect on SB, H2c: Social norms have a direct impact on SB, H2d: Social norms have an indirect effect on SB, H2e: Social support has a direct effect on SB, H2f: Social support has an indirect effect on SB, H2g: Walking environment has a direct effect on SB, H2h: Walking environment has an indirect effect on SB).

***Hypothesis 3***: Social function affects SB (H3a: Social function has a direct effect on SB).

To validate the constructed hypothesized model, this study will utilize data from community-based older adults with T2DM in mainland China and adopt the path analysis method. Importantly, the hypothesized model has not been validated.

To our knowledge, this study is the first to use a theory-driven approach to identify the determinants of SB and their underlying mechanisms in community-dwelling older adults with T2DM, filling gaps in existing research on the determinants of SB in this specific population [20, 21, 39]. Previous studies on SB determinants in older adults have mainly focused on the general older adults population and used single-level frameworks, lacking a systematic and theory-driven perspective. Building upon the Jorge’s conceptual model, this study employs the Behaviour Change Wheel (BCW) model to explore influencing factors and their interactive mechanisms across capability (physical function, pain, and cognitive function), opportunity (social isolation, walking environment, and social support), and motivation (social function) dimensions. By doing so, it offers a more comprehensive understanding of SB formation mechanisms in this specific population. Additionally,, given the limited SB intervention studies targeting older adults with T2DM in existing literature, as well as the weak theoretical foundation and lack of specificity in current intervention programs [40], this study is grounded in the Chinese context and follows the Intervention Mapping (IM) framework to develop a logic model of SB determinants for this group. This will establish a theoretical basis for future targeted intervention strategies. The research findings not only enhance theoretical understanding of the SB influencing mechanisms in older adults from a social-ecological perspective but also provide empirical references for the future development of effective interventions to reduce SB among community-dwelling older adults with T2DM, making significant contributions to both theory and practice.

## Method

### Study design

This study employed a cross-sectional research design to analyze the determinants of SB and their underlying mechanisms in community-dwelling older adults with T2DM. The study adhered to the Strengthening the Reporting of Observational Studies in Epidemiology (STROBE) guidelines for reporting [41].

### Data collection and ethical considerations

We recruited community-dwelling older adults diagnosed with T2DM at a diabetes management center located in Shandong, China, using a convenience sampling method from November 2021 to March 2022. This specific diabetes management center was selected due to its comprehensive platform for managing older adults with T2DM within the community. Additionally, the center’s staff could collect data from patients residing in multiple regions in northern China. This strategic choice facilitated our ability to comprehensively and accurately examine the factors associated with SB among community-dwelling older adults with T2DM.

The study received formal approval from the Ethics Committee of Beijing University of Chinese Medicine (approval number 2022BZYLL0505) and was conducted in accordance with the guidelines outlined in the Declaration of Helsinki. All participants were asked to independently complete a paper questionnaire after providing detailed verbal informed consent. Their responses were kept strictly confidential to ensure the participants’ privacy. The survey was completely anonymous and voluntary.

### Participants

The inclusion criteria for this study were as follows: (1) age ≥ 60 years, (2) hospital diagnosis of T2DM, and (3) permanent residents of the community (having lived in the region for more than six months); and (4) provided informed consent and participated voluntarily. The exclusion criteria were as follows: (1) communication disorders that prevented patients from completing the survey, (2) mental disorders, (3) physical disabilities that prevented participants from standing, and (4) the presence of other serious diseases, such as severe cardiac, hepatic, or renal insufficiency, respiratory failure, and malignant tumors.

The sample size for this study was determined using the cross-sectional survey sample size estimation formula $\text{n}={\left(\frac{{\mu }_{\alpha /2}\sigma }{\delta }\right)}^{2}$. We used data the survey conducted by Paing et al. [10] on the self-management behavior SB of patients with T2DM as a reference for calculating the sample size of the current study, which resulted in an estimated sample size of 480 cases. According to the requirement for sample size in structural equation modeling, the sample size should be at least 5 ~ 10 times the number of paths to be estimated in the model. In our preliminary model, we evaluated 45 items, so the minimum number of cases needed was 225 to 450. Therefore, the estimated sample size of 480 meets the sample size requirement for structural equation modeling.

### Measures

We used validated scales to assess SB, walking environments, social support, social isolation, social norms, social function, pain level, physical function, and cognitive function among patients. Additionally, we administered self-reported questionnaires to collect demographic information, including age, gender, Body Mass Index (BMI), educational attainment, marital status, residential status, and monthly personal income. Clinical characteristics such as disease duration, fasting glucose levels, and the presence of comorbid chronic illnesses were also examined.

### Sedentary behavior

To assess SB in community-dwelling older adults with T2DM, we developed the SB Questionnaire for older adults with T2DM (MOST-T2DM). The questionnaire development process included a literature review, expert consultation, and patient interviews to ensure the comprehensiveness and applicability of the questionnaire content. The MOST-T2DM questionnaire is based on two widely used and validated questionnaires: the Measure of Older Adults’ Sedentary Time (MOST) [42] and the Chinese version of the Sedentary Behavior Questionnaire for the older adults [31]. These two questionnaires showed good reliability, validity, and criterion association validity [31, 42]. The MOST-T2DM aims to measure the total daily sedentary time of older adults with T2DM over the past week and the amount of sedentary time that is spent engaging in six common aspects of daily life: screen time (e.g., watching TV), reading (e.g., reading books), socializing (e.g., talking on the phone), transportation (e.g., driving), hobbies (e.g., listening to music), and engaging in other activities that require sitting (e.g., eating). Sedentary time was categorized into 15-minute intervals.

The questionnaire was rigorously validated; analyses performed by four methodologists and four clinical nurse specialists indicated that the MOST-T2DM had excellent content validity (I-CVI: 1.000, with all responses being concordant; S-CVI: 1.000, mean S-CVI: 1.000). The test-retest reliability coefficients for the dimensions of screen time, reading, socializing, transportation, hobbies, engaging in other activities, and total sedentary time were 0.71, 0.99, 0.70, 0.70, 0.99, 0.98, and 0.50, respectively.

### Walking environment

The Abbreviated-Neighborhood Environment Walkability Scale (ANEWS), revised by Rena Zhou [43], was used to assess the walking environment of residents in urban communities in China. The scale comprises 17 items categorized into 5 dimensions: supportive facilities (4 items), street conditions (5 items), beautification (2 items), traffic (3 items), and safety (3 items). Each item is rated on a 5-point scale ranging from 1 (“very inconsistent”) to 5 (“very consistent”). The total score of the scale is the sum of scores for each item, and a higher score indicates a better walking environment. The Cronbach alpha coefficient of the scale was 0.807, the intragroup correlation coefficient (ICC) was 0.945, and the Spearman correlation coefficient was 0.721.

### Social support

The Social Support Rating Scale (SSRS) developed the Shuiyuan Xiao and colleagues, was utilized to evaluate social support [44]. The scale consisted of ten items across three dimensions: subjective support, objective support, and use of support. Subjective support encompassed four items (items 1, 3, 4, 5), objective support included three items (items 2, 6, 7), and use of support comprised three items (items 8, 9, 10). The total score was calculated by summing the scores of all ten items. Higher scores indicated greater levels of social support. The Cronbach alpha coefficient for the scale was 0.896, and the correlation coefficients between the three dimensions and the total scale ranged from 0.724 to 0.835, demonstrating good reliability and content validity [45]. This scale was employed in a previous study involving community-dwelling older adults with T2DM [46].

### Social isolation

The Lubben Social Network Scale 6 (LSNS-6) was utilized to evaluate the recent contact of older adults with family members and neighborhood friends. The scale consists of two dimensions, i.e., family and friends, with a maximum score of 30 points. A higher total score indicates stronger social ties among older adults, and a score below 12 points suggests social isolation. The English version of the LSNS-6 demonstrated good reliability, with a Cronbach alpha coefficient of 0.83 and content validity ranging from 0.68 to 0.78 [47]. In 2018, researchers in Hong Kong conducted a reliability analysis of the Chinese version of the LSNS-6 [48]. They found a better model fit (χ^2^ = 175.33, df = 8, CFI = 0.99, TLI = 0.98, RMSEA = 0.08) and a Cronbach alpha coefficient of 0.83 (0.90 for the family dimension and 0.95 for the friends dimension). This scale provides a more objective measure of social isolation among older adults individuals and is suitable for assessing social isolation in both medical fast-track environments and home care models. It has also been successfully applied to assess social isolation in community-dwelling older adults with T2DM [46].

### Social norm

Referring to the social norms-related items in the Determinants of Implementation Behavior Questionnaire (DIBQ) [49], the researcher administered the Subjective Social Norms Questionnaire for Sedentary Behavior (SSNQ-SB) to older adults with T2DM. The SSNQ-SB aimed to assess the impact of social norms on patients’ SB, focusing on family members, friends, and medical personnel. Participants rated their agreement on a 5-point scale, ranging from “strongly disagree” to “strongly disagree”. The higher the score is, the stronger the subjective norms, with a maximum score of 25. To ensure the questionnaire’s validity, 8 experts (including four methodologists and four clinical nurse specialists) assessed its content validity. Additionally, the questionnaire’s reliability was evaluated with 10 community-dwelling older adults with T2DM. The questionnaire was shown to have good content validity (I-CVI of 1.000 for each item, consistent S-CVI of 1,000 for all items, and mean S-CVI of 1.000) and good reliability (Cronbach’s alpha coefficient of 0.754).

### Social function

The Functional Activities Questionnaire (FAQ), developed by Pfeffer et al. in 1982 [50], was utilized to evaluate the current social function of older adults in the community. The FAQ comprises 10 items, including tasks such as using cards, paying for cards, shopping independently, engaging in skillful games or activities, using the stove, preparing meals, learning about new things, understanding attention, remembering essential appointments, and going out alone for activities or visiting friends. Each item is scored on a 3-point scale “0” indicates no difficulty and the task can be performed independently without any assistance, “1” indicates some difficulty and requires guidance or support from others, and “2” indicates inability to perform the task alone and instead relies on others for assistance. If an item is not applicable, it is marked as nine and not included in the scoring. The total score on the scale is 20, and a score greater than five or deficits in three or more functions may indicate a decline in social function. The scale is widely utilized and has demonstrated good reliability and validity [51].

### Pain

The Numerical Rating Scale (NRS) [52] was utilized to measure the intensity of pain using a scale of 0 to 10. A score of “0” indicated the absence of pain, while a score of “10” indicated the presence of severe pain. Mild pain was indicated by a score from 1 to 3, moderate pain was indicated by a score from 4 to 6, and severe pain was indicated by a score from 7 to 9. The NRS is known for its high reliability and ease of recording.

### Physical function

The Short Physical Performance Battery (SPPB) was utilized to evaluate physical function [53, 54]. The SPPB comprises three tests: balance, walking speed, and five times sit-to-stand. Each test is scored on a scale ranging from 0 to 4, resulting in a total SPPB score ranging from 0 to 12. A score of “0–6” indicates poor mobility, a score of “7–9” indicates moderate mobility, and a score of “10–12” indicates good mobility. The SPPB is a simple and convenient tool for assessing the physical function of older adults, particularly the mobility of their lower limbs. It has been widely adopted and utilized in many countries, including China.

### Cognitive function

The Montreal Cognitive Assessment Text, Beijing version (MoCA-BJ) [55, 56]was translated and revised by Wang et al. in 2007. It is based on the Montreal Cognitive Assessment Text (MoCA) [57] and is the most widely used version of the MoCA in China. The MoCA-BJ consists of seven scales that assess visuospatial and executive functions, naming, attention, language, abstraction, delayed memory, and orientation. The total score on the MoCA-BJ is 30 points, and a screening cutoff value of 26 is used in the original version. A test result is considered normal if the score is greater than 26. The MoCA-BJ is designed to screen 90% of mildly cognitively impaired individuals and is a simplified version of the scale.

#### Data collation

(1) Eliminate unqualified questionnaires. Questionnaires that were incomplete or had more than 10% of the questions omitted were excluded. Additionally, questionnaires with the same selected items in the self-reported section were also eliminated. (2) Data entry: An Excel database was established to enter and organize the data. A real-time double-entry check was performed to ensure the accuracy of data entry. (3) Data verification: After completing the data entry, a logical check was conducted to identify any errors. The original information was consulted for correction to ensure the quality of data entry, specifically addressing logical errors.

#### Data analysis

The statistical analysis was conducted using SPSS 25.0 and Amos 26.0, with a predetermined significance level of *α* = 0.05. The methodology employed in the study is outlined in Table 1. Path analysis (PA) was used to determine the path structure and establish model hypotheses, drawing upon prior theoretical frameworks and literature. The dataset included variables related to SB, the quality of the walking environment, social isolation, adherence to social norms, social support, social function, pain perception, physical function, and cognitive function. The model fit was estimated, and the statistical significance of path coefficients was evaluated. If the model exhibited suboptimal fit, corrective measures were taken. These adjustments were guided by the significance of the pathways, modification indices, and alignment with theoretical foundations, ensuring a more robust and scientifically rigorous model.

**Table 1:** Selection of statistical analysis methods

Analysis of content	Analyzing indicators	Statistical analysis methods
Description of current situation	Age, gender, BMI, education, marital status, mode of residence, monthly personal income, disease duration, fasting glucose, comorbid chronic diseases, SB, walking environment, social isolation, social norms, social function, pain, physical function, cognitive function	Mean ± standard deviation Frequency (composition ratio)
Comparing levels of SB in patients with different general profiles	Age, gender, BMI, education, marital status, mode of residence, personal monthly income, duration of illness, fasting blood glucose, presence of comorbid chronic diseases, SB	Independent t-test analysis of variance (ANOVA)
Clarify the role of determinants related to SB	SB, walking environment, social isolation, social norms, social function, pain, physical function, cognitive function	Path analysis

#### Quality control

The design phase of this study involved conducting a literature review, selecting reliable measurement tools, and consulting with experts to address potential issues. Trained researchers were responsible for the data collection phase, where they distributed questionnaires to study participants and explained the study’s purpose, obtaining consent. The collected data were carefully checked and corrected during the data collection process. Data entry was performed using real-time double-entry checking to ensure accuracy. Additionally, logical checks and corrections were made after the data entry was completed.

## Results

### Participant characteristics

We initially recruited 500 participants aged 60 or older with T2DM and administered questionnaires to all of them. After excluding 11 invalid questionnaires (six with the same options, three who were unwilling to continue due to temporary matters, and two who had privacy concerns), a total of 489 (97.80%) questionnaires were obtained for analysis. The average age of the respondents was 69.13 ± 3.94 years. Most of the patients were overweight or obese. The majority had a junior high school education level (44.99%). Furthermore, most of the older adults with T2DM were married (80.78%) and not living alone (91.62%). The average duration of the disease was 16.02 ± 6.39 years. Table 2 included details on the characteristics of the respondents.

**Table 2:** Characteristics of study participants (*N* = 489)

Characteristic	Categories	Number of cases(%)	Sedentary time(h/d)$\bar{x}\pm s$	F/t	*P*
Age	60–69	249 (50.92)	6.41 ± 1.38	−15.180^a^	0.000*
	≥ 70	240 (49.08)	8.22 ± 1.27		
Sex	Male	236 (48.26)	7.35 ± 1.58	0.713^a^	0.476
	Female	253 (51.74)	7.25 ± 1.63		
BMI (kg/m^2^)	≤ 18.5	10 (2.04)	7.13 ± 1.28	0.148^b^	0.931
	18.5–23.9	196 (40.08)	7.28 ± 1.67		
	24.0–27.9	212 (43.35)	7.34 ± 1.55		
	≥ 28.0	71 (14.52)	7.23 ± 1.65		
Education Level	Primary School or Below	137 (28.02)	7.76 ± 1.78	7.555^b^	0.000*
	Junior Middle School	220 (44.99)	7.29 ± 1.37		
	Senior High School or Vocational School	99 (20.25)	6.96 ± 1.63		
	College/Associate Degree or Higher	33 (6.75)	6.42 ± 1.66		
Marital Status	Married	395 (80.78)	7.28 ± 1.62	−0.499^a^	0.618
	Other	94 (19.22)	7.37 ± 1.54		
Residential Status	Living Alone	41 (8.38)	7.84 ± 1.15	3.033^a^	0.004*
	Not Living Alone	448 (91.62)	7.25 ± 1.63		
Income (RMB/Month)	≤ 1999	206 (42.13)	7.63 ± 1.62	15.436^b^	0.000*
	2000–3999	154 (31.49)	7.32 ± 1.40		
	4000–5999	99 (20.25)	7.08 ± 1.50		
	≥ 6000	30 (6.13)	5.64 ± 1.73		
Coexistence of Chronic Condition	Yes	386 (78.94)	7.65 ± 1.44	10.481^a^	0.000*
	No	103 (21.06)	5.97 ± 1.49		
Duration of Illness in Years	≤ 5	27 (5.52)	6.32 ± 1.47	30.312^b^	0.000*
	6–15	202 (41.31)	6.78 ± 1.48		
	16–25	223 (45.60)	7.62 ± 1.53		
	≥ 26	37 (7.57)	8.90 ± 1.11		
Fasting Blood Glucose (mmol/L)	≤ 6.9	163 (33.33)	7.43 ± 1.47	1.351^a^	0.178
	≥ 7.0	326 (66.67)	7.23 ± 1.67		

*Note*: ^a^: independent samples *t*-test; ^b^: ANOVA, analysis of variance; *: *P* < 0.05;RMB: renminbi, we conducted the survey in China and collected data in RMB (Renminbi, the official currency of the People’s Republic of China)

## Current status of SB in community-dwelling older adults with T2DM

### Levels of SB in community-dwelling older adults with T2DM

The study found that community-dwelling older adults diagnosed with T2DM reported an average of 7.30 ± 1.61 h of SB per day. Notably, 80.37% of the participants reported being sedentary for over six hours per day, and 37.01% reported greater than eight hours of SB per day. These findings highlight the high prevalence of SB among older adults with T2DM, as shown in Table 3.

**Table 3:** Levels of SB in community-dwelling older adults with T2DM (h/d)

Index	Minimum	Maximum	Median	*P*_25_	*P*_75_
Screen Class	0.00	6.00	2.75	2.00	3.50
Reading	0.00	1.00	0.00	0.00	0.00
Socializing	0.00	3.50	1.00	0.50	1.50
Transportation	0.00	1.50	0.00	0.00	0.50
Hobbies	0.00	4.00	1.50	0.50	2.00
Other activities that require sitting	0.50	5.75	1.50	1.00	2.00

### Comparison of levels of SB in community-dwelling older adults with T2DM across different characteristics

Statistically significant differences in sedentary time were observed among community-dwelling older adults with T2DM based on various characteristics, such as age groups, literacy levels, living arrangements, income levels, disease duration, and the presence of other chronic diseases (all with *P* < 0.05). However, no statistically significant differences in sedentary time were detected based on sex, BMI, marital status, or fasting glucose levels (all with *P* > 0.05) (see Appendix 2).

## Validation of a model for determinants associated with SB in community-dwelling older adults with T2DM

### Current status of potential determinants of SB in community-dwelling older adults with T2DM

In this study, we employed the ANEWS, SSRS, LSNS-6, and SSNQ-SB to measure the levels of the walking environment, social support, social isolation, and subjective social norms, respectively, among community-dwelling older adults with T2DM. Our findings showed that all four indicators were, on average, at an intermediate level (ANEWS total score = 57.69 ± 5.64, SSRS total score = 40.56 ± 5.44, LSNS-6 total score = 16.53 ± 2.86, SSNQ-SB mean score = 16.36 ± 3.44). We also used the SPPB and FAQ to evaluate the physical and social functions, respectively, of these patients. The results indicated good overall levels of functioning, with average total scores of 10.18 ± 1.02 for the SPPB and 2.49 ± 1.26 for the FAQ. Additionally, we used the NRS to assess pain levels among older adults with T2DM living in the community, and the average NRS score was 2.61 ± 1.39, indicating a mild overall pain level. The relevant outcome data can be found in Appendix 3.

### Preliminary validation of a model for determinants associated with SB in community-dwelling older adults with T2DM

SB was used as the outcome variable, which encompassed six dimensions: screen time, reading, socializing, using transportation, hobbies, and other sitting activities. The sum of these dimensions indicated the level of SB. Considering the sample size of this study and the skewness and kurtosis coefficients of each variable, the maximum likelihood (ML) method was selected as the model evaluation method.

### Model fitness assessment

After testing, the preliminary model is a saturated model (SM), resulting in a unique solution for the model estimate calculation. The sample moment has 45 independent elements, which corresponds to the number of data points that the sample data covariance matrix can provide. The model requires estimating 45 individual parameters and has a degree of freedom value of 0, a chi-square value of 0, and the probability value *P* for the chi-square significance test cannot be calculated. However, importantly, saturated models often lack practical guidance value in real-life situations and may require further revision.

### Path coefficients for direct effects in the preliminary model

**Fig. 2:**
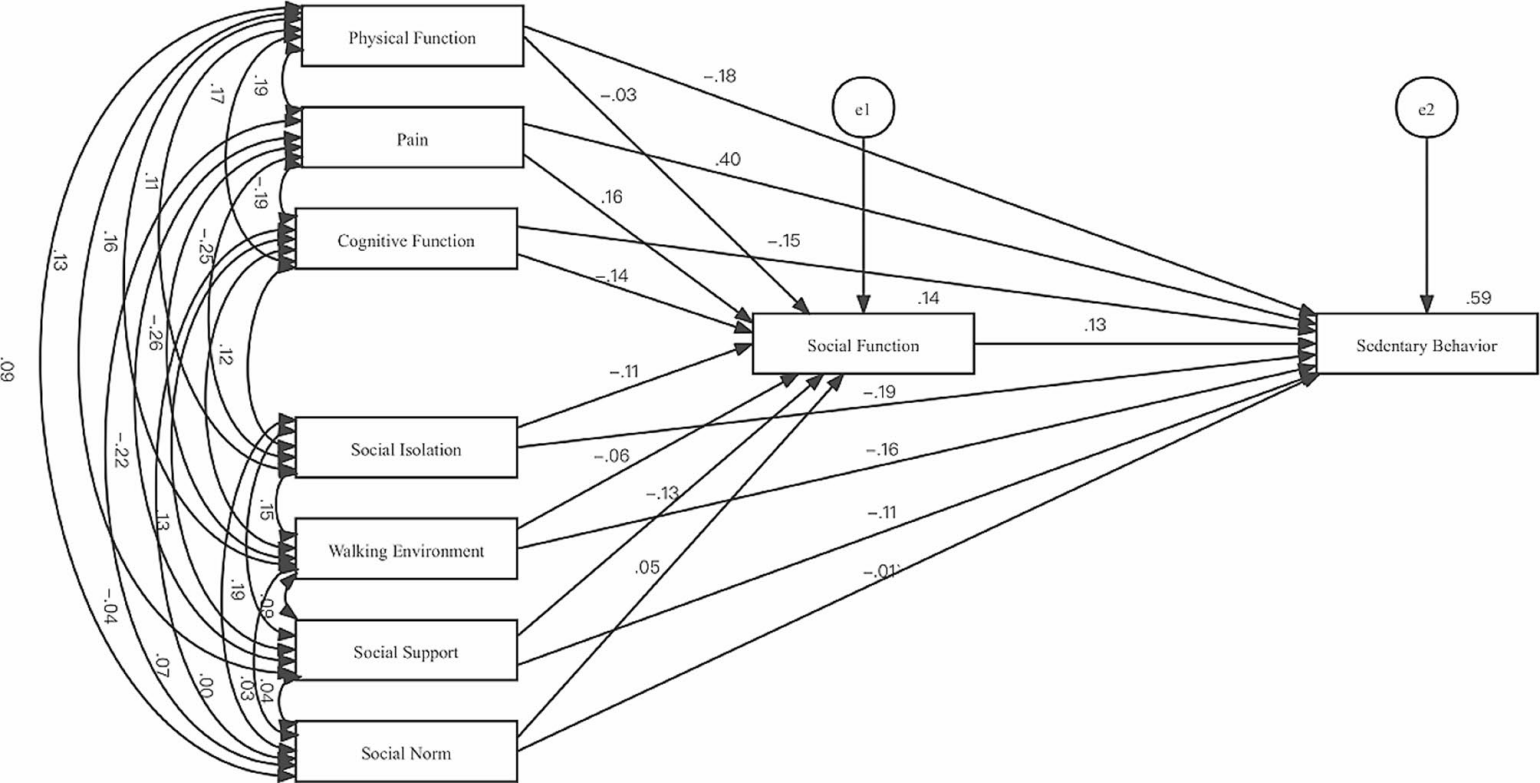
The relationship diagram for preliminary model validation

Table 4 presents the path coefficients and test results for the direct effect of each factor. Additionally, Fig. 2 illustrates the model relationship diagram. Out of all the paths tested, 11 direct effect paths exhibited statistical significance (*P* ＜ 0.05), while four paths did not show statistical significance.

Based on the model fitness and path coefficient test results mentioned above, it is evident that the initial model requires further revision considering the theoretical and literature bases.

**Table 4:** Estimated and tested path coefficients for the direct effect of each factor

From	To	Standardized coefficient	C.*R*.	*P*
Cognitive function	Social function	−0.137	−3.151	0.002
Social support	Social function	−0.128	−2.912	0.004
Social norm	Social function	0.046	1.078	0.281
Physical Function	Social function	−0.033	−0.748	0.455
Pain	Social function	0.165	3.573	***
Social isolation	Social function	−0.109	−2.471	0.013
Walking environment	Social function	−0.056	1.265	0.206
Social function	Sedentary behavior	0.127	4.065	***
Walking environment	Sedentary behavior	−0.163	−5.326	***
Social support	Sedentary behavior	−0.111	−3.618	***
Social isolation	Sedentary behavior	−0.194	−6.288	***
Physical Function	Sedentary behavior	−0.179	−5.893	***
Pain	Sedentary behavior	0.402	12.454	***
Cognitive function	Sedentary behavior	−0.152	−4.991	***
Social norm	Sedentary behavior	−0.008	−0.271	0.786

*Note*: ***: *P* < 0.001

### Model modification of determinants associated with SB in community-dwelling older adults with T2DM

We made corrections by considering the significance of the paths, correction indices (with a threshold value > 3.84) [58, 59], and relevant literature. Nonsignificant paths were removed, and each deleted path underwent iterative testing until all direct interaction paths between variables in the model achieved statistical significance. The eliminated paths included social norms to social function, physical function to social function, walking environment to social function, and social norms to SB.

### Final model fit assessment

The overall fitness of the final tested model is shown in Table 5. The indicators of model fit were as follows: root mean square error of approximation (RMSEA) = 0.014, chi-squared ratio of degrees of freedom (*χ*^2^/*df*) = 1.100, GFI = 0.999, AGFI = 0.980, NFI = 0.997, RFI = 0.954, IFI = 1.000, TLI = 0.996, and CFI = 1.000. All these indicators exceed the threshold of 0.90, indicating that the model meets the standards for fitness. Therefore, the overall assessment of model fitness is good.

**Table 5:** Values of final model fitness index

Fitness Indices	Fitness Standards	Fitness Values
Absolute Fit Indices		
*χ*^*2*^	*P* > 0.05	2.201(*P* = 0.333)
RMSEA values	< 0.08	0.014
GIF values	> 0.9	0.999
AGFI values	> 0.9	0.980
Incremental Fitness Indices		
NFI	> 0.9	0.997
RFI	> 0.9	0.954
IFI	> 0.9	1.000
TLI	> 0.9	0.996
GFI	> 0.9	1.000
Parsimony Fitness Indices		
*χ* ^*2*^ */df*	< 2.00	1.100

### Path coefficients for the direct effects of factors in the final model

**Fig. 3:**
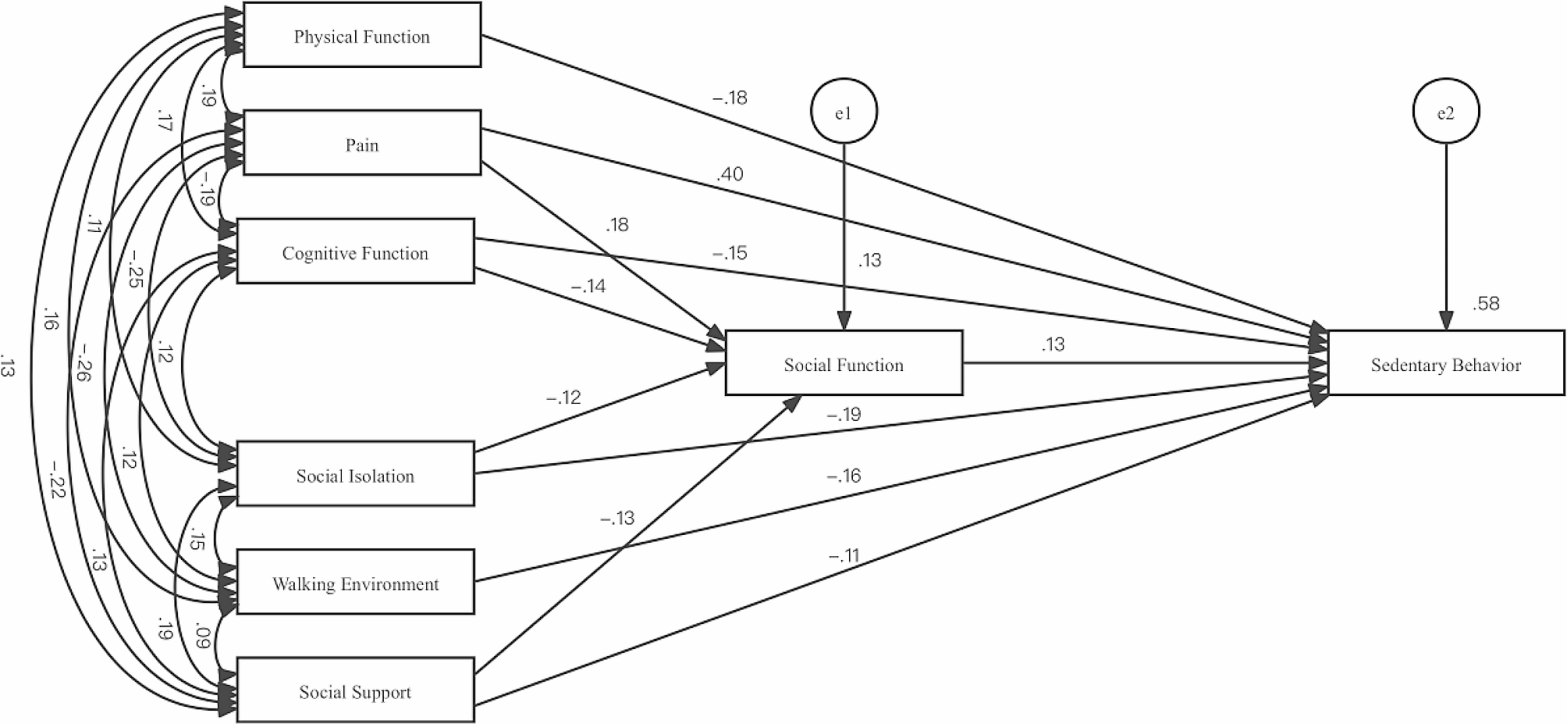
The modified path model of the relationship between SB and its determinants

Based on Jorge’s conceptual model of SB in older adults and the conceptual model established by the BCW, the final model diagram (Fig. 3) includes social support, pain, social isolation, walking environment, social function, physical function, cognitive function, and the outcome variable SB. The direct effects of each factor, along with their path coefficients and test results, are presented in Table 6. Additionally, Table 7 summarizes the total, direct, and indirect effects of each path. The model results provide insights into the relationship between the determinants of SB in older adults with T2DM.

**Table 6:** Estimated path coefficients and tests for direct effects of factors

From	To	Standardized Coefficients	C.*R*.	*P*
Cognitive function	Social function	−0.142	−3.290	0.001
Social support	Social function	−0.130	−2.960	0.003
Pain	Social function	0.180	4.010	***
Social isolation	Social functionn	−0.117	−2.635	0.008
Social function	Sedentary behavior	0.127	4.065	***
Walking environment	Sedentary behavior	−0.163	−5.339	***
Social support	Sedentary behavior	−0.111	−3.625	***
Social isolation	Sedentary behavior	−0.194	−6.280	***
Physical function	Sedentary behavior	−0.180	−5.931	***
Pain	Sedentary behavior	0.403	12.429	***
Cognitive function	Sedentary behavior	−0.153	−5.009	***

*Note*: ***= *P* < 0.05

The relationship between capability and SB was examined in older adults with T2DM. Table 6 presents the data indicating that physical function, pain, and cognitive function have an impact on SB in older adults with T2DM. Among these factors, physical function and cognitive function have a greater influence on SB than on pain. Physical function directly affects SB in older adults with T2DM (standardized coefficient: -0.180). Additionally, pain and cognitive function directly affected SB in these patients (standardized coefficients: 0.403 and − 0.153, respectively). Moreover, they also indirectly influence SB through social functions (standardized coefficients: 0.023 and − 0.018, respectively).

The relationship between opportunities and SB in older adults with T2DM is influenced by the walking environment, social isolation, and social support. Social isolation has a slightly stronger impact on SB than the walking environment and social support. Social isolation directly affects SB in older adults with T2DM (standardized coefficient of -0.194). Alternatively, it can indirectly affect SB through social function (standardized coefficient of -0.015). The walking environment also has a direct influence on SB in older adults with T2DM (standardized coefficient of -0.163). Similarly, social support may directly impact SB in older adults with T2DM (standardized coefficient of -0.111) and indirectly affect SB through social function (standardized coefficient of -0.017).

The relationship between social function and SB in older adults with T2DM can be directly influenced by the social function, as indicated by a standardized coefficient of 0.127.

## Discussion

This study examined the prevalence of SB among community-dwelling older adults with T2DM in China. By utilizing the BCW framework and conducting a literature review, the researchers determined the factors that contribute to SB in Chinese older adults with T2DM. The study identified the effects of capability, social function, and opportunity on SB and elucidated the underlying mechanisms. The model developed in this study indicated that pain and cognitive function directly and indirectly impact SB in older adults with T2DM, while physical function directly affects SB. Moreover, opportunity-related factors such as social isolation and social support have both direct and indirect effects on SB, whereas the walking environment has only a direct effect on sedentary behavior. Last, the motivation domain is influenced by social function, which directly affects patients’ SB. These findings provide strong evidence for the proposed relationships among the variables studied.

**Table 7:** Direct, indirect, and total effects of factors on SB

Factors	Direct Effects		Indirect Effects		Total Effects Standardized Coefficients
	Paths	Standardized Coefficients	Paths	Standardized Coefficients	
Physical Function	Physical Function→ Sedentary behavior	−0.180*	-	-	−0.180*
Pain	Pain→Sedentary behavior	0.403*	Pain→ Social function→ Sedentary behavior	0.023*	0.426*
Cognitive function	Cognitive function→ Sedentary behavior	−0.153*	Cognitive function→ Social function→ Sedentary behavior	−0.018*	−0.171*
Social isolation	Social isolation→ Sedentary behavior	−0.194*	Social isolation→ Social function→ Sedentary behavior	−0.015*	−0.209*
Walking environment	Walking environment→ Sedentary behavior	−0.163*	-	-	−0.163*
Social support	Social support→Sedentary behavior	−0.111*	Social support→ Social function→ Sedentary behavior	−0.017*	−0.128*
Social function	Social function→Sedentary behavior	0.127*	-	-	0.127*

*Note*:* *P* < 0.05;the Bootstrap was used to test mediation

### Analysis of SB levels in community-dwelling older adults with T2DM

In this study, we examined the SB of community-dwelling older adults with T2DM. On average, these patients spent 7.30 ± 1.61 h per day being sedentary. We found that 80.37% of the patients were sedentary for more than six hours per day, and 37.01% were sedentary for more than eight hours per day. Our findings showed a slightly higher average daily sedentary time than the results reported in previous studies on SB among community-dwelling older adults with T2DM in China. For example, the average daily sedentary time reported herein exceeded that reported by Du Jin et al. [8]. An average daily sedentary time of 7.01 ± 2.15 h was reported in a study of 426 older adults aged 60 years or older with T2DM in Zhengzhou City.

Similarly, our results differed from previous studies on SB levels in community-dwelling older adults with T2DM, which reported averages ranging from 6.25 ± 1.59 to 6.88 ± 1.98 h/d [60, 61]. However, our findings were lower than the SB levels observed in older adults residing in nursing homes, which averaged 8.85 ± 2.81 h per day [49]. Although our observed average daily sedentary time was higher than the studies by Cooper et al. [13] and Paing et al. [10] (8.0 ± 1.2 h/d and 9.8 ± 1.8 h/d, respectively), it was lower than the SB levels reported by Nakanishi et al. [18] and Chen et al. [12] (4 h/d and 3.4 ± 2.5 h/d, respectively). These variations highlight the wide variability in SB levels among community-dwelling older adults with T2DM, which can be influenced by factors such as geographical location, context, study population, measurement timing, and assessment tools. Our study found that 80.37% of community-dwelling older adults with T2DM averaged more than 6 h per day of SB, with 37.01% exceeding 8 h per day, indicating a prevalent pattern of SB in this population.

## Model analysis of the role of determinants related to SB in community-dwelling older adults with T2DM in SB

### The relationship between capability and SB

#### The role of physical function in SB

The results of this study suggest that there is a direct relationship between physical function and SB in older patients with T2DM. The standardized coefficient of -0.180 indicates that poorer physical function is associated with longer sedentary times. These findings support the findings of Jason J. Wilson et al. [62], who also found a significant association between longer sedentary time and poorer physical function in a study involving 1,360 community-dwelling older adults (*P* < 0.05). Another study [63], which included 1,168 adult patients with knee osteoarthritis, found that less sedentary time was linked to better physical function in patients with knee osteoarthritis. Specifically, the group with the longest sedentary time had a mean gait speed of 1.18 m/s, which was significantly lower than that of the groups with less sedentary time (1.29, 1.32, and 1.32 m/s, respectively). Additionally, the group with the longest sedentary time had a notably lower mean stance velocity (25.9 stands/minute) than the group with the shortest sedentary time (28.9, 29.1, and 31.1 n/min, respectively). These trends remained statistically significant even after adjusting for demographic factors, health-related variables, and the average daily duration of moderate-to-vigorous physical activity.

#### The role of pain in SB

In this study, we discovered that pain has a direct and indirect impact on the SB of community-dwelling older adults with T2DM. Essentially, higher levels of pain in these individuals are associated with longer sedentary times. On the one hand, pain can directly influence the SB of these patients, as indicated by a standardized coefficient of 0.403. This finding aligns with a study conducted by De Souza et al. [64], which revealed associations between prolonged sedentary time and neck pain (OR = 2.09; 95% CI: 1.08, 4.04), upper back pain (OR = 2.21; 95% CI: 1.07, 4.56), and lumbar pain (OR = 1.91; 95% CI: 1.00, 3.53). Furthermore, a cross-sectional survey by Da et al. [65] involving 1,011 adolescents found that neck pain in girls was associated with moderate SB (OR = 1.80; 95% CI: 1.00, 3.23) and high SB (OR = 1.91; 95% CI: 1.02, 3.53), while neck pain in boys was associated with moderate SB (OR = 2.75; 95% CI: 1.31, 5.78). Girls also showed associations between low back pain and moderate SB (OR = 2.73; 95% CI: 1.45, 5.02) and very high SB (OR = 2.49; 95% CI: 1.30, 4.76). A systematic review and meta-analysis by Baradaran et al. [66], comprising 49 observational studies, concluded that office workers who experience low levels of back pain have a higher likelihood of engaging in SB (OR = 1.23). Similar findings were reported among older adults with osteoarthritis [67], indicating that individuals spend more time sitting in the morning when they experience heightened pain, resulting in decreased engagement in moderate-to-vigorous physical activity during the day. Additionally, cross-day lag analyses demonstrated that morning pain has a lasting effect on subsequent SB, with longer sedentary periods associated with increased pain the following morning. These studies collectively suggest that reducing pain exacerbation may contribute to a decrease in SB and an increase in physical activity levels. Beyond its direct impact on SB, pain may indirectly influence SB by affecting social function. This is supported by a standardized coefficient of 0.023. Hengstebeck et al. [68] conducted a study involving 966 respondents and revealed that chronic pain could impair work ability by interfering with social function. Importantly, prosocial behavior and positive interpersonal interactions can have a positive influence on individuals’ daily routines, thereby improving SB and physical activity levels.

#### The role of cognitive function in SB

The results of this study indicate that cognitive function in community-dwelling older adults with T2DM may have a direct or indirect influence on SB. Specifically, cognitive function was found to have a direct influence on SB in older adults with T2DM (standardized coefficient of 0.153). These findings are consistent with a study by Wanders et al. [69], which examined the relationship between sedentary time and cognitive function in 2821 participants. In the final model, sedentary time during activities such as television watching, reading, or engaging in creative pursuits was not associated with cognitive function (all *P* > 0.05). However, a significant positive association was observed between total sedentary time and cognitive function in a diverse population. This association varied across different domains, with sedentary time related to work and computer use showing a particularly strong positive association with cognitive function. These findings suggest that the relationship between sedentary time and cognitive function may differ depending on the specific domain. Another study [70] reported that a 1-unit decrease in cognitive function scores was associated with a 2% increase in SB (*P* ≤ 0.01). Additionally, every 1-unit decrease in lower extremity strength, as measured by chair standing scores, was found to increase SB by 5% points (*P* ≤ 0.01). The most significant increase in SB was observed in older adults with decreased cognitive function and concomitant changes in lower extremity strength. The study by Rojer et al. [71] in 2021 examined the relationship between SB and cognitive function in older adults. The systematic review included 45 publications with a total of 15,817 older patients. The findings from longitudinal studies (*n* = 7) indicated that higher levels of moderate-to-vigorous and light physical activity, a long period with lower levels of SB, were associated with better overall cognitive function. Similarly, the results from the included cross-sectional study (*n* = 38) showed that less SB was associated with better cognitive function. Importantly, cognitive function can also indirectly influence SB through social function (standardized coefficient of -0.018). Cognitive function plays a significant role in maintaining activities of daily living and the social dimensions of aging [72]. Another study by Han et al. [73] suggested a positive correlation between cognitive function and social function, thus highlighting the independent influence of cognitive function on social function. Therefore, cognitive function may have an impact on SB in community-dwelling T2DM patients by affecting social function.

### The relationship between opportunity and SB

#### The role of the walking environment in SB

In this study, the influence of the walking environment on the SB of community-dwelling older adults with T2DM was examined (standardized coefficient of -0.163). The factors that were found to directly influence SB included supportive amenities, street conditions, landscaping, transportation, and policing. This finding is consistent with the study by Brach et al. [74], which suggested that adults living in a planned group home environment with supportive services had higher levels of SB than those living in private homes. Importantly, the living environment of older adults can contribute to their SB. Despite living in communities, community-dwelling older adults reside in diverse housing environments which can have an impact on their SB. Another study by Chang et al. [75] also supported these findings, showing that increased availability of sidewalks was associated with a decrease in the number and duration of sedentary episodes. This study specifically focused on older Taiwanese adults and highlighted the importance of a good community environment, characterized by the presence of sidewalks, in reducing SB. These findings have significant implications for the development of environmental policies aimed at reducing SB among older adults.

#### The role of social isolation in SB

Based on the findings of this study, it has been determined that social isolation has a direct or indirect impact on the SB of community-dwelling older adults with T2DM. Caruso et al. [76] conducted a study to examine the occurrence of loneliness, SB, and falls in older adults before and during the COVID-19 pandemic, particularly during periods of social isolation. The study revealed a significant increase in loneliness and SB during social isolation, although no increase in falls was observed. Furthermore, social isolation may indirectly influence SB through its effect on social function (standardized coefficient of -0.015). When older adults with T2DM who reside in the community experience social isolation, their level of social function decreases, resulting in an increase in sedentary time.

#### The role of social support in SB

The findings of this study suggest that social support has a direct or indirect impact on SB, particularly in older adults with T2DM. The direct influence of social support on SB was found to have a standardized coefficient of -0.111. Social support can be provided in various forms, including emotional, informational, and pathway support, and can come from medical staff, family members, friends, and peers. Adequate social support, such as providing knowledge about SB, guidance on physical activity, or organizing group activities such as walking or dancing, can significantly improve the daily lives of older adults and help reduce SB. Furthermore, social support indirectly affects SB by influencing social function (standardized coefficient of 0.017). Peer support in disease self-management has long been recognized as important for patients with T2DM [77, 78].

### The relationship between motivation (social function) and SB

In this study, the researchers found that social function has a direct impact on SB in older patients with T2DM (standardized coefficient of 0.127). Improved social function leads to increased engagement in various activities, such as using cards, paying bills, shopping independently, participating in skillful games or activities, using the stove, preparing meals, learning about new things, understanding attention, remembering essential appointments, and going out on one’s own or visiting friends. These social activities can occupy a significant portion of an older person’s time, serving as an alternative to SB. This suggests that enabling older adults to fully utilize their social function enhances their sense of self-worth, brings about lifestyle changes, and reduces negative emotions associated with social isolation. Furthermore, it also promotes different levels of physical activity and reduces SB.

In addition, the impact of social norms on SB, as discovered in this study, has not yet reached statistical significance. This finding contradicts the results of Howlett et al. [79], who observed that subjective norms can present opportunities to influence SB. This discrepancy could be attributed to variations in the populations studied and inconsistencies in the instruments employed to measure subjective criteria. Consequently, further research is needed to explore the influence of social norms on SB.

### Inspiration of theoretical models

This study, based on a literature review by Jorge’s conceptual model of determinants of sedentary behavior in older adults, along with the the Behavior Change Wheel (BCW), examined the factors influencing SB among community-dwelling older adults with T2DM in China. The findings partially validate Jorge’s model, suggesting that sedentary behavior in older adults is shaped by personal, social, and environmental factors. However, the influence of these factors varies, with personal factors such as pain, social isolation, physical function, and cognitive function playing more significant roles in SB among community-dwelling older adults with T2DM. This finding is consistent with the BCW’s emphasis on individual psychological and behavioral aspects such as capability and motivation [80].

Nevertheless, the study also revealed that social functional factors (such as social norms) had a limited influence on the SB of this population. This challenges Jorge’s model’s theoretical premise that social norms are a significant determinant of SB in older adults and highlights potential limitations of the BCW model in explaining the socioecological determinants of personal behavior at the meso and macrolevels. This suggests that understanding and intervening in the SB of older adults with T2DM in communities may require an integration of different theoretical perspectives and a dynamic consideration of the interactions between individuals and their environments. The Systems of Sedentary Behaviours (SOS) framework offers a novel approach [81]. Unlike the BCW model, which focuses more on individual-level factors, the SOS framework emphasizes the complexity and multidimensionality of SB, adopting a systems theory-based interdisciplinary analytical perspective that examines the interactive effects of determinants of SB from the individual to public policy across various dimensions. The SOS categorizes the determinants of SB into six systems: physical health and wellbeing, social and cultural context, psychology and behaviour, politics and economics, and institutional and home settings.

The SOS framework underscores the interconnectedness of different systems and elements, yet it offers relatively less discussion on the specific interaction mechanisms between determinants. By examining older adults with T2DM in Chinese communities, this study explored the intrinsic mechanisms of these determinants, thereby enhancing our understanding of the complex mechanisms underlying SB. Moreover, the SOS framework emphasizes the importance of interdisciplinary collaboration and the adoption of multilevel intervention strategies. Our findings also support this view, as the factors influencing the SB of older adults with T2DM involve multiple domains including physical health, mental health, and the social environment. This implies that designing intervention strategies requires comprehensive consideration of these factors and an interdisciplinary approach.

Future research could further explore the application of the SOS framework in guiding intervention strategies for SB among older adults with T2DM, as well as how it can be integrated with other theoretical models such as the BCW framework to better understand and intervene in this population’s SB. A deeper exploration of the integration of different theoretical perspectives will help to more fully understand the complexity of SB, providing theoretical guidance and practical references for effectively improving the health status of older adults with T2DM.

## Limitations of the study

This study has several limitations. First, the sample selection was limited to hospitals in northern cities of mainland China with type 2 diabetes management programs, which may restrict the generalizability of the findings. Second, the survey instrument relied primarily on subjective self-reports rather than objective biomarkers or medical records.This reliance on self-reports may introduce recall bias, particularly for events or behaviors that occurred in the past. Therefore, future studies should consider incorporating additional objective data collection methods to enhance the reliability and validity of the results. Due to limitations in our study conditions, we were unable to additionally validate the structural validity and discriminant validity of the MOST-T2DM questionnaire. Future research will consider employing a variety of validation methods to further refine the psychometric properties of the MOST-T2DM questionnaire. Moreover, although this study utilized the BCW framework, which effectively reveals the intrinsic connections between determinants of SB, it may not fully capture the richness and subtleties of these determinants. In this context, Ecological Momentary Analysis (EMA) provides a method for real-time data collection in natural settings and is particularly suited for capturing the complexity of behavioral and sociocognitive determinants on a microtime scale. This method can be used to assess fluctuations in physical activity-related behaviors and their determinants across time and space [82]. Therefore, future studies might consider integrating the BCW framework with EMA to capture the dynamic nature of SB and comprehensively consider physical activity behaviors (including varying intensities of physical activity, sedentary time, and sleep) and their determinants. This integrated approach will help deepen the understanding of the multifaceted determinants affecting sedentary behavior, thereby providing a theoretical basis for devising effective intervention strategies. Finally, due to the cross-sectional correlational analyses employed in this study, it is not possible to definitively determine whether the identified factors have a causal influence on SB among older adults with type 2 diabetes in the community. Future research should consider employing longitudinal study designs or experimental methods to test behavioral interventions aimed at enhancing capabilities, providing opportunities, and stimulating motivation (social function) to reduce SB among community-dwelling older adults with T2DM. This approach would allow for a deeper understanding of these causal relationships and their potential moderating mechanisms. Additionally, qualitative research can offer deeper insights and complement findings from quantitative studies. In this study, determinants related to capabilities, opportunities, and motivation (social function) could only explain only 58% of the variance in sedentary behavior, suggesting that additional influencing factors remain to be explored. Qualitative studies in different populations have identified factors such as knowledge, mental health, and social pressures as influencing sedentary behavior [83, 84]. Therefore, future research could further employ qualitative methodologies to intricately explore the lived experiences and personal challenges associated with SB among community-dwelling older adults with T2DM.

## Conclusions

This study systematically explored potential mechanisms associated with SB and its determinants among older adults with T2DM in a Chinese community. We integrated the BCW with a literature review to illuminate the intricate factors. Our findings indicate that capability factors (physical function, pain perception, and cognitive function), opportunity factors (social isolation, the walking environment, and social support), and social function factors are all significant predictors of SB in older adults with T2DM. Notably, we observed that pain, social isolation, physical function, cognitive function, the walking environment, social support, and social function may impact SB in decreasing order of influence. Building upon these insights, we have developed a comprehensive conceptual model outlining SB in community-dwelling older adults with T2DM. However, the significance and variability of the factors in our study model were inconsistent. For instance, while the walking environment is important, it is difficult to change; in contrast, social support is not only important but also highly variable. To address this issue, we will assign changeability scores to these factors to guide the development of intervention strategies.

### Electronic supplementary material

Below is the link to the electronic supplementary material.

Supplementary Material 1

Supplementary Material 2

Supplementary Material 3

Supplementary Material 4

## Data Availability

The datasets used and analyzed during the current study are available from the corresponding author on reasonable request. This study was approved by the Ethics Committee of Beijing University of Chinese Medicine (approval number 2022BZYLL0505). Written informed consent was obtained from participants. Written informed consent was obtained from participants for publication. The authors declare that they have no competing interests.

## Abbreviations

T2DM: Type 2 Diabetes Mellitus
SB: Sedentary Behavior
BCW: Behaviour Change Wheel
COM-B: Capability-Opportunity-Motivation-Behavior
TDF: Theoretical Domain Framework
