# Supplementary material for: Determinants of sedentary behavior in community-dwelling older adults with type 2 diabetes based on the behavioral change wheel: a path analysis
Source: BMC Geriatr. 2024 Jun 6;24:502. doi: 10.1186/s12877-024-05076-0 (PMC11157943; doi:10.1186/s12877-024-05076-0)
Supplement: Supplementary file 2 — Supplementary Material 2 [file 12877_2024_5076_MOESM2_ESM.docx]

**Appendix 1**

Definitions and constructs of Opportunity Motivation - Behaviour (COM-B) and Theoretical Domains Framework (TDF), as well as the links between COM-B and Theoretical Domains TDF. Source: Nilsen, P., & Birken, S. A. (Eds.). (2020). Handbook on Implementation Science. Edward Elgar Publishing Limited. [https://doi.org/10.4337/9781788975995](https://doi.org/10.4337/9781788975995)

| **COM-B components** | | **Theoretical domains**  **framework domains** | **Definition** | **Theoretical concepts portrayed in each domain** |
| --- | --- | --- | --- | --- |
| **Capability** | **Psychological capability** | Knowledge | An awareness of the existence of something | Knowledge (including knowledge of condition/scientific rationale), procedural knowledge, knowledge of task environment |
|  |  | Behavioral regulation | Anything aimed at managing or changing objectively observed or measured actions | Self-monitoring, breaking habit, action planning |
|  |  | Memory, attention, and decision processes | The ability to retain information, focus selectively on aspects of the environment and choose between two or more alternatives | Memory, attention, attention control, decision- making, cognitive overload/tiredness |
|  | **Physical capability** | Skills | An ability of or proficiency acquired through practice | Skills, skills development, competence, ability, practice, skill assessment |
| **Motivation** | **Reflective motivation** | Social/professional roles and identities | A coherent set of behaviors and displayed personal qualities of an individual in a social or work setting | Professional identity, professional role, social identity, identity, professional boundaries, professional confidence, group identify, leadership, organizational commitment |
|  |  | Beliefs about capabilities | Acceptance of the truth, reality, or validity about an ability, talent, or facility that a person can put to constructive use | Self-confident, perceived competence, self-efficacy, perceived behavioral control, beliefs, self-esteem, empowerment, professional confidence |
|  |  | Optimism | The confidence that things will happen for the best or that desired goals will be attained | Optimism, pessimism, unrealistic optimism, identity |
|  |  | Beliefs about consequences | Acceptance of the truth, reality, or validity about outcomes of a behavior in a given situation | Beliefs, outcome expectancies, characteristics of outcome expectancies, anticipated regret, consequents |
|  |  | Intentions | A conscious decision to perform a behavior or a resolve to act in a certain way | Stability of intentions, stages of change model, transtheoretical model and stages of change |
|  |  | Goals | Mental representations of outcomes or end states that an individual wants to achieve | Goals (distal/proximal), goal priority, goal/target setting, goals (autonomous/controlled), action planning, implementation intention |
|  | **Automatic motivation** | Reinforcement | Increasing the probability of a response by arranging a dependent relationship, or contingency, between the response and a given stimulus | Rewards (proximal/distal, valued/not values, probable/improbable), incentives, punishment, consequents, reinforcement, contingencies, sanctions |
|  |  | Emotion | A complex reaction pattern, involving experiential, behavioral, and physiological elements, by which the individual attempts to deal with a personally significant matter or event | Fear, anxiety, affect, tress, depression, positive/negative affect, burn-out |
| **Opportunity** | **Physical opportunity** | Environmental context and resources | Any circumstance of a person’s situation or environment that discourages or encourages the development of skills and abilities, independence, social competence, and adaptive behavior | Environmental stressors, resources/material resources, organizational culture/climate, salient events/critical incidents, person x environment interaction, barriers and facilitators |
|  | **Social opportunity** | Social influences | Those interpersonal processes that can cause individuals to change their thoughts, feeling, or behaviors | Social pressure, social norms, group conformity, social comparisons, groups norms, social support, power, intergroup conflict, alienation, group identity, modelling |
