# Supplementary material for: Determinants of sedentary behavior in community-dwelling older adults with type 2 diabetes based on the behavioral change wheel: a path analysis
Source: BMC Geriatr. 2024 Jun 6;24:502. doi: 10.1186/s12877-024-05076-0 (PMC11157943; doi:10.1186/s12877-024-05076-0)
Supplement: Supplementary file 3 — Supplementary Material 3 [file 12877_2024_5076_MOESM3_ESM.docx]

**Appendix 2**

**Table 1 Comparison of sedentary behavior in** **older adults T2DM patients with different characteristics**

| **Characteristic** | **Categories** | **Number of cases（%）** | **Sedentary time（h/d）**  **$\bar{x}\pm s$** | ***F*/*t*** | ***P*** |
| --- | --- | --- | --- | --- | --- |
| Age | 60~69 | 249 ( 50.92 ) | 6.41 ± 1.38 | −15.180^a^ | 0.000^*^ |
|  | ≥ 70 | 240 ( 49.08 ) | 8.22 ± 1.27 |  |  |
| Sex | Man | 236 ( 48.26 ) | 7.35 ± 1.58 | 0.713^a^ | 0.476 |
|  | Female | 253 ( 51.74 ) | 7.25 ± 1.63 |  |  |
| BMI  （kg/m^2^） | ≤ 18.5 | 10 ( 2.04 ) | 7.13 ± 1.28 | 0.148^b^ | 0.931 |
|  | 18.5 < BMI < 24 | 196 ( 40.08 ) | 7.28 ± 1.67 |  |  |
|  | 24 ≤ BMI < 28 | 212 ( 43.35 ) | 7.34 ± 1.55 |  |  |
|  | ≥ 28.0 | 71 ( 14.52 ) | 7.23 ± 1.65 |  |  |
| Education Level | Primary School or Below | 137 ( 28.02 ) | 7.76 ± 1.78 | 7.555^b^ | 0.000^*^ |
|  | Junior Middle School | 220 ( 44.99 ) | 7.29 ± 1.37 |  |  |
|  | Senior High School or Vocational School | 99 ( 20.25 ) | 6.96 ± 1.63 |  |  |
|  | College/Associate Degree or Higher | 33 ( 6.75 ) | 6.42 ± 1.66 |  |  |
| Marital Status | Married | 395 ( 80.78 ) | 7.28 ± 1.62 | −0.499^a^ | 0.618 |
|  | Other | 94 ( 19.22 ) | 7.37 ± 1.54 |  |  |
| Residential Status | Living Alone | 41 ( 8.38 ) | 7.84 ± 1.15 | 3.033^a^ | 0.004^*^ |
|  | Not Living Alone | 448 ( 91.62 ) | 7.25 ± 1.63 |  |  |
| Income (RMB/Month) | ≤ 1999 | 206 ( 42.13 ) | 7.63 ± 1.62 | 15.436^b^ | 0.000^*^ |
|  | 2000~3999 | 154 ( 31.49 ) | 7.32 ± 1.40 |  |  |
|  | 4000~5999 | 99 ( 20.25 ) | 7.08 ± 1.50 |  |  |
|  | ≥ 6000 | 30 ( 6.13 ) | 5.64 ± 1.73 |  |  |
| Coexistence of Chronic Condition | Yes | 386 ( 78.94 ) | 7.65 ± 1.44 | 10.481^a^ | 0.000^*^ |
|  | No | 103 ( 21.06 ) | 5.97 ± 1.49 |  |  |
| Duration of Illness in Years | ≤ 5 | 27 ( 5.52 ) | 6.32 ± 1.47 | 30.312^b^ | 0.000^*^ |
|  | 6~15 | 202 ( 41.31 ) | 6.78 ± 1.48 |  |  |
|  | 16~25 | 223 ( 45.60 ) | 7.62 ± 1.53 |  |  |
|  | ≥ 26 | 37 ( 7.57 ) | 8.90 ± 1.11 |  |  |
| Fasting Blood Glucose （mmol/L） | ≤ 6.9 | 163 ( 33.33 ) | 7.43 ± 1.47 | 1.351^a^ | 0.178 |
|  | ≥ 7.0 | 326 ( 66.67 ) | 7.23 ± 1.67 |  |  |

Note: ^a^：independent samples *t*-test; ^b^：ANOVA, analysis of variance; *: *P* < 0.05；RMB: renminbi, we conducted the survey in China and collected data in RMB (Renminbi, the official currency of the People's Republic of China).
