# Supplementary material for: Determinants of sedentary behavior in community-dwelling older adults with type 2 diabetes based on the behavioral change wheel: a path analysis
Source: BMC Geriatr. 2024 Jun 6;24:502. doi: 10.1186/s12877-024-05076-0 (PMC11157943; doi:10.1186/s12877-024-05076-0)
Supplement: Supplementary file 4 — Supplementary Material 4 [file 12877_2024_5076_MOESM4_ESM.docx]

**Appendix 3**

**Current status of potential determinants of SB in community-dwelling** **older adults with T2DM**

**Table 1** Scores of community-dwelling older adults with T2DM on ANEWS

| **Item** | **Minimum** | **Maximum** | **$\bar{x}\pm s$** | **Score range** |
| --- | --- | --- | --- | --- |
| ANEWS total score | 38 | 75 | 57.69 ± 5.64 | 17 ~ 85 |
| Supportive facilities | 8 | 20 | 14.12 ± 2.88 | 4 ~ 20 |
| Street conditions | 5 | 25 | 14.39 ± 3.54 | 5 ~ 25 |
| Beautification | 2 | 10 | 5.62 ± 1.72 | 2 ~ 10 |
| Traffic | 7 | 15 | 11.05 ± 1.87 | 3 ~ 15 |
| Safety | 10 | 15 | 12.51 ± 1.23 | 3 ~ 15 |

**Table 2** The SSRS scores in community-dwelling older adults with T2DM

| **Item** | **Minimum** | **Maximum** | **$\bar{x}\pm s$** | **Score range** |
| --- | --- | --- | --- | --- |
| SSRS total score | 24 | 55 | 40.56 ± 5.44 | 12 ~ 64 |
| Subjective support | 11 | 28 | 22.10 ± 3.18 | 8 ~ 32 |
| Objective support | 5 | 15 | 10.28 ± 1.87 | 1 ~ 20 |
| Use of support | 3 | 12 | 8.18 ± 1.76 | 3 ~ 12 |

**Table 3** LSNS-6 scores in community-dwelling older adults with T2DM

|  | **Minimum** | **Maximum** | **$\bar{x}\pm s$** | **Score range** |
| --- | --- | --- | --- | --- |
| LSNS-6 total score | 8 | 25 | 16.53 ± 2.86 | 0 ~ 30 |
| Family dimension | 4 | 13 | 8.36 ± 2.14 | 0 ~ 15 |
| Friend dimension | 4 | 12 | 8.17 ± 1.82 | 0 ~ 15 |

**Table 4** SPPB scores in community-dwelling older adults with T2DM

|  | **Minimum** | **Maximum** | **$\bar{x}\pm s$** | **Score range** |
| --- | --- | --- | --- | --- |
| SPPB total score | 7 | 12 | 10.18 ± 1.02 | 0 ~ 12 |
| Balance | 2 | 4 | 3.38 ± 0.67 | 0 ~ 4 |
| Walking speed | 2 | 4 | 3.47 ± 0.61 | 0 ~ 4 |
| Sit-to-stand | 2 | 4 | 3.33 ± 0.67 | 0 ~ 4 |

**Table 5** FAQ scores community-dwelling older adults with T2DM

|  | **Minimum** | **Maximum** | **Median** | **$\bar{x}\pm s$** | **Score range** |
| --- | --- | --- | --- | --- | --- |
| FAQ total score | 0 | 5 | 0 | 2.49 ± 1.26 | 0~20 |
| 1. Using cards | 0 | 2 | 0 | 0.20 ± 0.43 | 0~2 |
| 1. Paying for cards | 0 | 2 | 0 | 0.36 ± 0.52 | 0~2 |
| 1. Shopping independently | 0 | 2 | 0 | 0.37 ± 0.52 | 0~2 |
| 1. Engaging in skillful games or activities | 0 | 2 | 0 | 0.26 ± 0.48 | 0~2 |
| 1. Using the stove | 0 | 2 | 0 | 0.26 ± 0.50 | 0~2 |
| 1. Preparing meals, | 0 | 2 | 0 | 0.29 ± 0.51 | 0~2 |
| 1. Learning about new things | 0 | 2 | 0 | 0.23 ± 0.45 | 0~2 |
| 1. Understanding attention | 0 | 2 | 0 | 0.25 ± 0.46 | 0~2 |
| 1. Remembering essential appointments | 0 | 2 | 0 | 0.15 ± 0.38 | 0~2 |
| 1. Going out alone for activities or visiting friends | 0 | 2 | 0 | 0.11 ± 0.33 | 0~2 |

**Table 6** SSNQ-SB scores in community-dwelling older adults with T2DM

| **Item** | **Minimum** | **Maximum** | **$\bar{x}\pm s$** | **Score range** |
| --- | --- | --- | --- | --- |
| 1. My friends think I should not sit for too long | 1 | 5 | 3.54 ± 0.92 | 1~5 |
| 1. My family thinks I should not sit for too long | 1 | 5 | 3.52 ± 0.96 | 1~5 |
| 1. Medical staff think I should not sit for too long | 2 | 5 | 3.79 ± 0.71 | 1~5 |
| 1. My friends do not sit for too long | 1 | 5 | 2.80 ± 1.10 | 1~5 |
| 1. My family did not have any sedentary behaviour | 1 | 5 | 2.72 ± 1.19 | 1~5 |

**Table 7** MoCA-BJ scores in community-dwelling older adults with T2DM

| **Item** | **Minimum** | **Maximum** | **$\bar{x}\pm s$** | **Score range** |
| --- | --- | --- | --- | --- |
| MoCA-BJ | 14 | 30 | 25.19 ± 2.52 | 0~30 |
| Visuospatial and executive functions | 1 | 5 | 3.35 ± 0.84 | 0~5 |
| Naming | 1 | 3 | 2.90 ± 0.31 | 0~3 |
| Attention | 2 | 6 | 4.31 ± 0.91 | 0~6 |
| Language | 2 | 3 | 2.72 ± 0.45 | 0~3 |
| Abstraction | 0 | 2 | 1.81 ± 0.40 | 0~2 |
| Delayed memory | 1 | 5 | 3.35 ± 0.99 | 0~5 |
| Orientation | 4 | 6 | 5.81 ± 0.40 | 0~6 |
